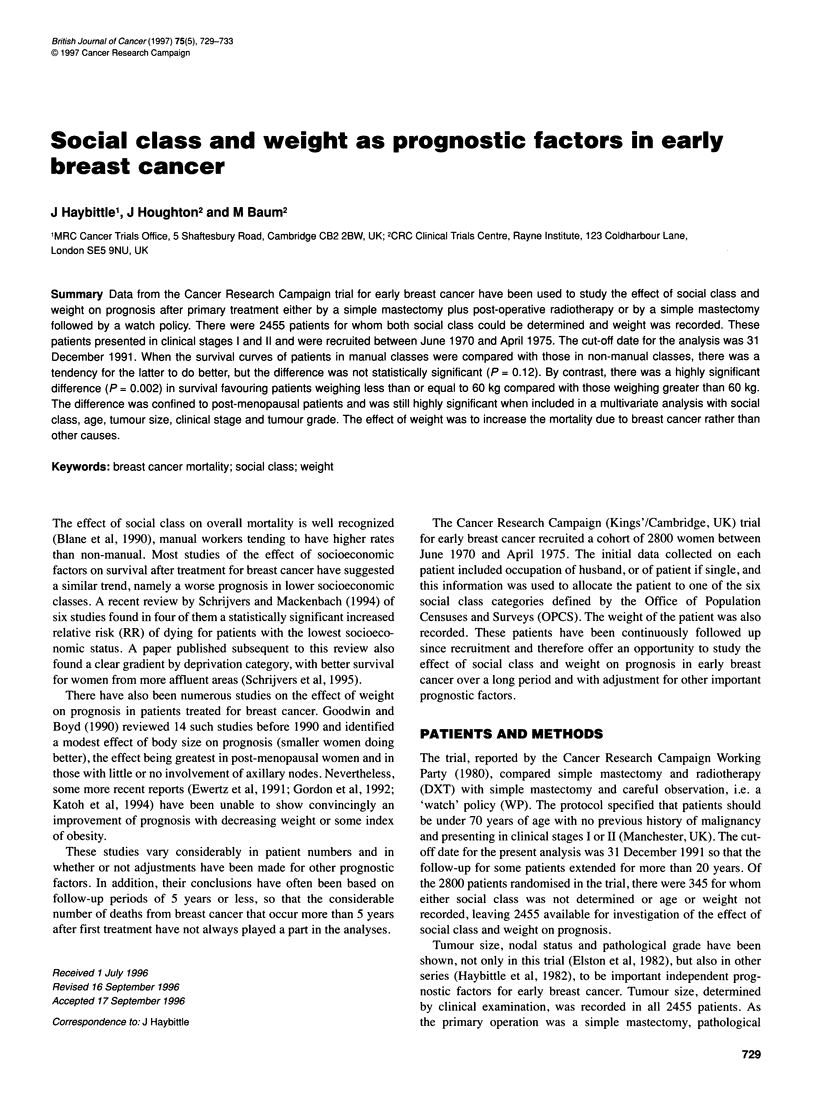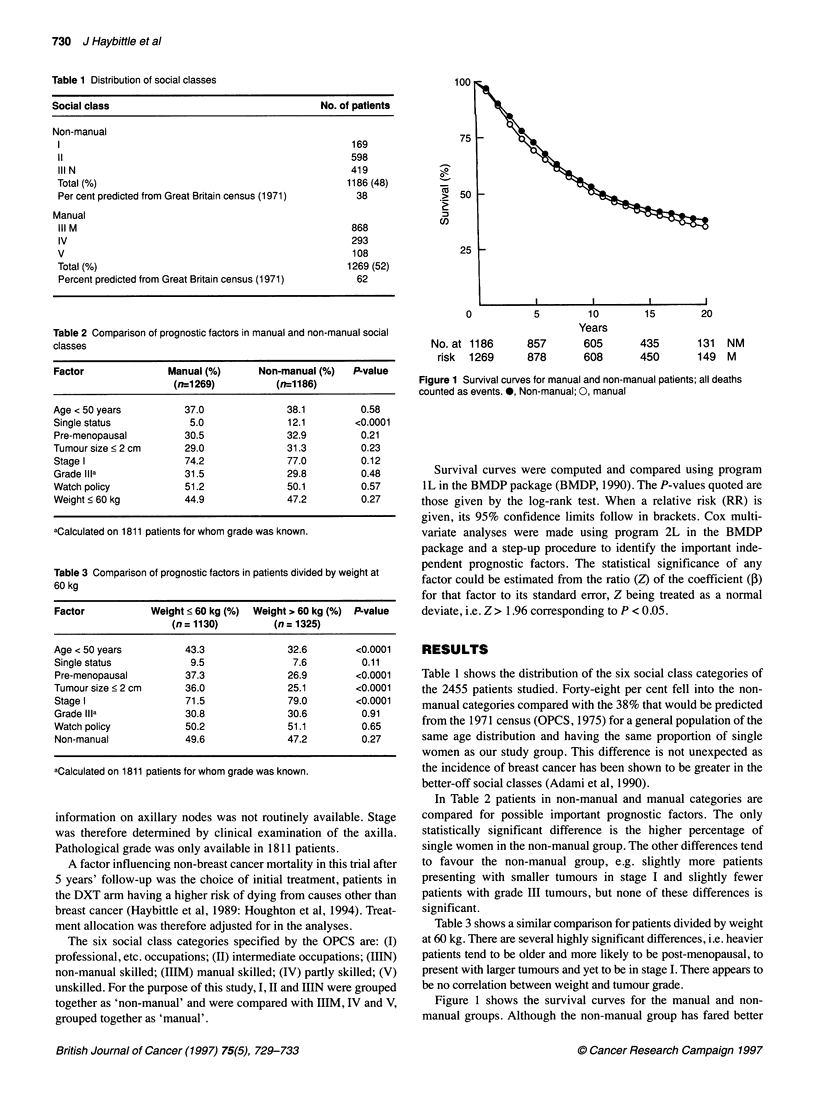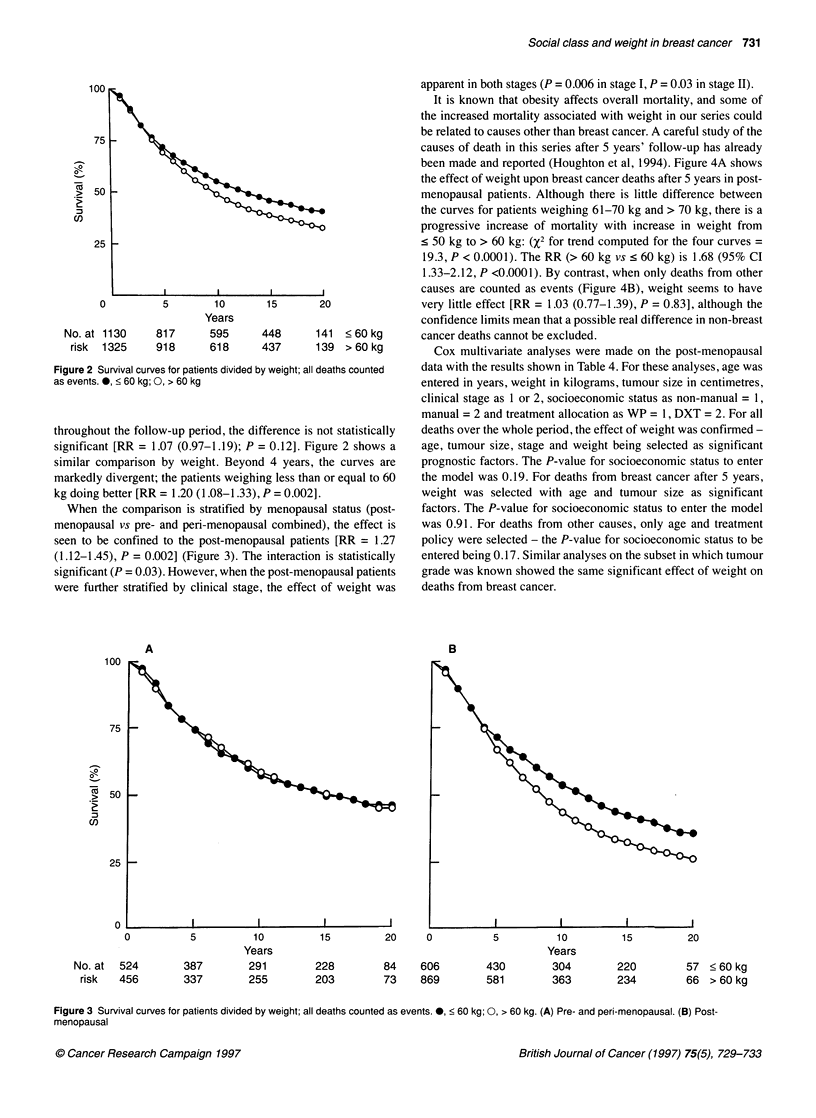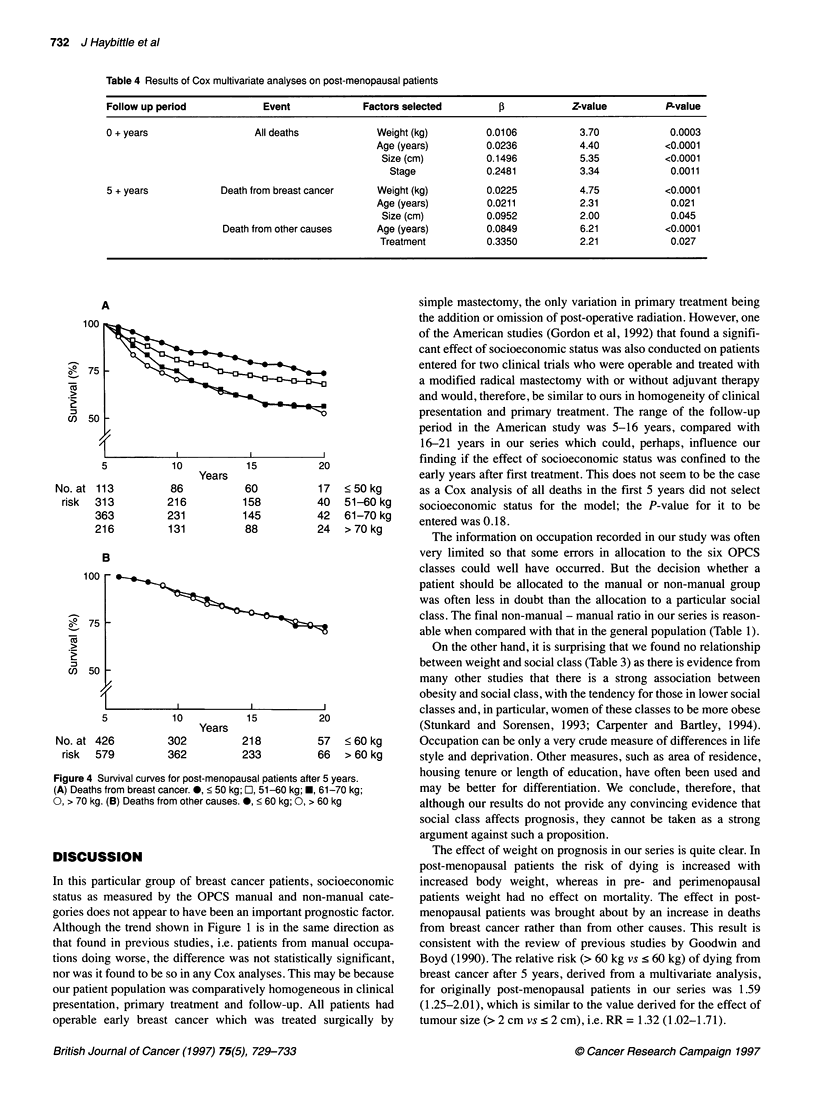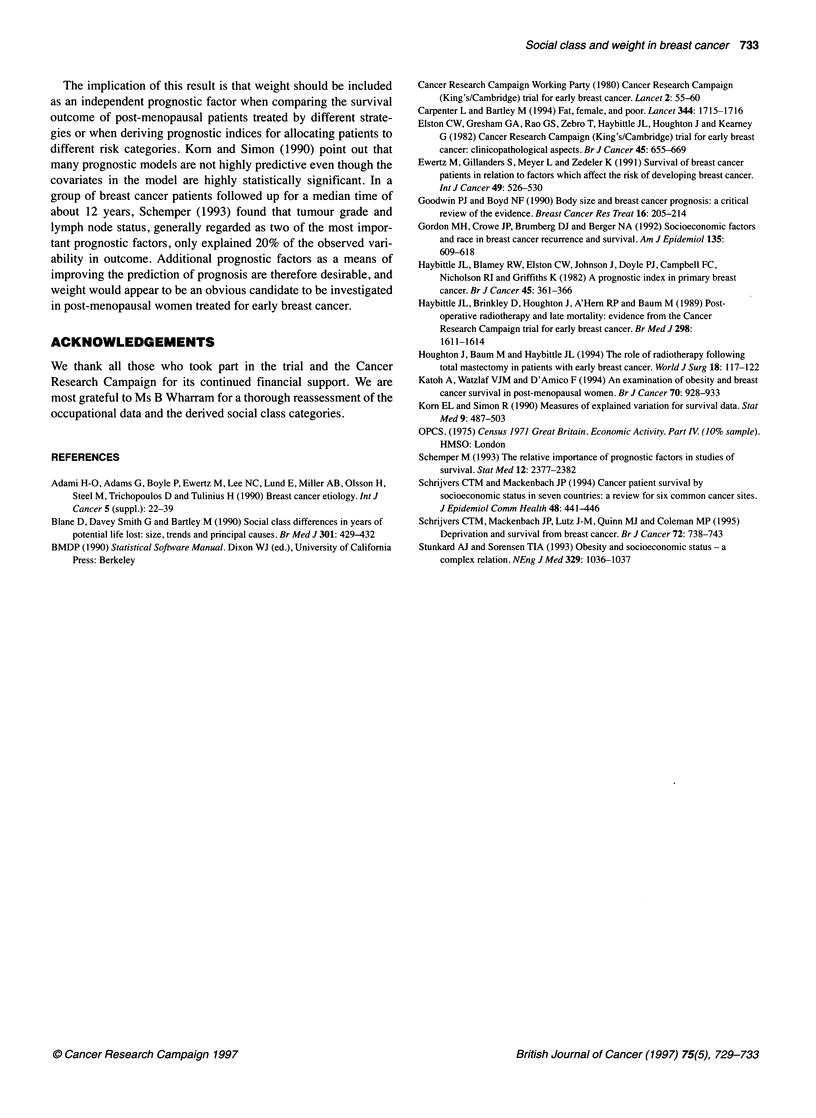# Social class and weight as prognostic factors in early breast cancer.

**DOI:** 10.1038/bjc.1997.129

**Published:** 1997

**Authors:** J. Haybittle, J. Houghton, M. Baum

**Affiliations:** MRC Cancer Trials Office, Cambridge, UK.

## Abstract

Data from the Cancer Research Campaign trial for early breast cancer have been used to study the effect of social class and weight on prognosis after primary treatment either by a simple mastectomy plus post-operative radiotherapy or by a simple mastectomy followed by a watch policy. There were 2455 patients for whom both social class could be determined and weight was recorded. These patients presented in clinical stages I and II and were recruited between June 1970 and April 1975. The cut-off date for the analysis was 31 December 1991. When the survival curves of patients in manual classes were compared with those in non-manual classes, there was a tendency for the latter to do better, but the difference was not statistically significant (P = 0.12). By contrast, there was a highly significant difference (P = 0.002) in survival favouring patients weighing less than or equal to 60 kg compared with those weighing greater than 60 kg. The difference was confined to post-menopausal patients and was still highly significant when included in a multivariate analysis with social class, age, tumour size, clinical stage and tumour grade. The effect of weight was to increase the mortality due to breast cancer rather than other causes.


*British Journal of Cancer* (1997) **75**(5), 729–733
© 1997 Cancer Research Campaign

### Social class and weight as prognostic factors in early breast cancer

**J Haybittle[1], J Houghton[2] and M Baum[2]**

[1]MRC Cancer Trials Office, 5 Shaftesbury Road, Cambridge CB2 2BW, UK; [2]CRC Clinical Trials Centre, Rayne Institute, 123 Coldharbour Lane, London SE5 9NU, UK

**Summary** Data from the Cancer Research Campaign trial for early breast cancer have been used to study the effect of social class and weight on prognosis after primary treatment either by a simple mastectomy plus post-operative radiotherapy or by a simple mastectomy followed by a watch policy. There were 2455 patients for whom both social class could be determined and weight was recorded. These patients presented in clinical stages I and II and were recruited between June 1970 and April 1975. The cut-off date for the analysis was 31 December 1991. When the survival curves of patients in manual classes were compared with those in non-manual classes, there was a tendency for the latter to do better, but the difference was not statistically significant ($P = 0.12$). By contrast, there was a highly significant difference ($P = 0.002$) in survival favouring patients weighing less than or equal to 60 kg compared with those weighing greater than 60 kg. The difference was confined to post-menopausal patients and was still highly significant when included in a multivariate analysis with social class, age, tumour size, clinical stage and tumour grade. The effect of weight was to increase the mortality due to breast cancer rather than other causes.

**Keywords:** breast cancer mortality; social class; weight

The effect of social class on overall mortality is well recognized (Blane et al, 1990), manual workers tending to have higher rates than non-manual. Most studies of the effect of socioeconomic factors on survival after treatment for breast cancer have suggested a similar trend, namely a worse prognosis in lower socioeconomic classes. A recent review by Schrijvers and Mackenbach (1994) of six studies found in four of them a statistically significant increased relative risk (RR) of dying for patients with the lowest socioeconomic status. A paper published subsequent to this review also found a clear gradient by deprivation category, with better survival for women from more affluent areas (Schrijvers et al, 1995).

There have also been numerous studies on the effect of weight on prognosis in patients treated for breast cancer. Goodwin and Boyd (1990) reviewed 14 such studies before 1990 and identified a modest effect of body size on prognosis (smaller women doing better), the effect being greatest in post-menopausal women and in those with little or no involvement of axillary nodes. Nevertheless, some more recent reports (Ewertz et al, 1991; Gordon et al, 1992; Katoh et al, 1994) have been unable to show convincingly an improvement of prognosis with decreasing weight or some index of obesity.

These studies vary considerably in patient numbers and in whether or not adjustments have been made for other prognostic factors. In addition, their conclusions have often been based on follow-up periods of 5 years or less, so that the considerable number of deaths from breast cancer that occur more than 5 years after first treatment have not always played a part in the analyses.

*Received 1 July 1996*
*Revised 16 September 1996*
*Accepted 17 September 1996*

*Correspondence to:* J Haybittle

The Cancer Research Campaign (Kings'/Cambridge, UK) trial for early breast cancer recruited a cohort of 2800 women between June 1970 and April 1975. The initial data collected on each patient included occupation of husband, or of patient if single, and this information was used to allocate the patient to one of the six social class categories defined by the Office of Population Censuses and Surveys (OPCS). The weight of the patient was also recorded. These patients have been continuously followed up since recruitment and therefore offer an opportunity to study the effect of social class and weight on prognosis in early breast cancer over a long period and with adjustment for other important prognostic factors.

### PATIENTS AND METHODS

The trial, reported by the Cancer Research Campaign Working Party (1980), compared simple mastectomy and radiotherapy (DXT) with simple mastectomy and careful observation, i.e. a 'watch' policy (WP). The protocol specified that patients should be under 70 years of age with no previous history of malignancy and presenting in clinical stages I or II (Manchester, UK). The cut-off date for the present analysis was 31 December 1991 so that the follow-up for some patients extended for more than 20 years. Of the 2800 patients randomised in the trial, there were 345 for whom either social class was not determined or age or weight not recorded, leaving 2455 available for investigation of the effect of social class and weight on prognosis.

Tumour size, nodal status and pathological grade have been shown, not only in this trial (Elston et al, 1982), but also in other series (Haybittle et al, 1982), to be important independent prognostic factors for early breast cancer. Tumour size, determined by clinical examination, was recorded in all 2455 patients. As the primary operation was a simple mastectomy, pathological

**Table 1** Distribution of social classes

| Social class | No. of patients |
|---|---|
| Non-manual | |
| I | 169 |
| II | 598 |
| III N | 419 |
| Total (%) | 1186 (48) |
| Per cent predicted from Great Britain census (1971) | 38 |
| Manual | |
| III M | 868 |
| IV | 293 |
| V | 108 |
| Total (%) | 1269 (52) |
| Percent predicted from Great Britain census (1971) | 62 |

**Table 2** Comparison of prognostic factors in manual and non-manual social classes

| Factor | Manual (%) (*n*=1269) | Non-manual (%) (*n*=1186) | *P*-value |
|---|---|---|---|
| Age < 50 years | 37.0 | 38.1 | 0.58 |
| Single status | 5.0 | 12.1 | <0.0001 |
| Pre-menopausal | 30.5 | 32.9 | 0.21 |
| Tumour size ≤ 2 cm | 29.0 | 31.3 | 0.23 |
| Stage I | 74.2 | 77.0 | 0.12 |
| Grade III[a] | 31.5 | 29.8 | 0.48 |
| Watch policy | 51.2 | 50.1 | 0.57 |
| Weight ≤ 60 kg | 44.9 | 47.2 | 0.27 |

[a]Calculated on 1811 patients for whom grade was known.

**Table 3** Comparison of prognostic factors in patients divided by weight at 60 kg

| Factor | Weight ≤ 60 kg (%) (*n* = 1130) | Weight > 60 kg (%) (*n* = 1325) | *P*-value |
|---|---|---|---|
| Age < 50 years | 43.3 | 32.6 | <0.0001 |
| Single status | 9.5 | 7.6 | 0.11 |
| Pre-menopausal | 37.3 | 26.9 | <0.0001 |
| Tumour size ≤ 2 cm | 36.0 | 25.1 | <0.0001 |
| Stage I | 71.5 | 79.0 | <0.0001 |
| Grade III[a] | 30.8 | 30.6 | 0.91 |
| Watch policy | 50.2 | 51.1 | 0.65 |
| Non-manual | 49.6 | 47.2 | 0.27 |

[a]Calculated on 1811 patients for whom grade was known.

information on axillary nodes was not routinely available. Stage was therefore determined by clinical examination of the axilla. Pathological grade was only available in 1811 patients.

A factor influencing non-breast cancer mortality in this trial after 5 years' follow-up was the choice of initial treatment, patients in the DXT arm having a higher risk of dying from causes other than breast cancer (Haybittle et al, 1989: Houghton et al, 1994). Treatment allocation was therefore adjusted for in the analyses.

The six social class categories specified by the OPCS are: (I) professional, etc. occupations; (II) intermediate occupations; (IIIN) non-manual skilled; (IIIM) manual skilled; (IV) partly skilled; (V) unskilled. For the purpose of this study, I, II and IIIN were grouped together as 'non-manual' and were compared with IIIM, IV and V, grouped together as 'manual'.

| | 0 | 5 | 10 | 15 | 20 | |
|---|---|---|---|---|---|---|
| No. at | 1186 | 857 | 605 | 435 | 131 | NM |
| risk | 1269 | 878 | 608 | 450 | 149 | M |

**Figure 1** Survival curves for manual and non-manual patients; all deaths counted as events. ●, Non-manual; ○, manual

Survival curves were computed and compared using program 1L in the BMDP package (BMDP, 1990). The *P*-values quoted are those given by the log-rank test. When a relative risk (RR) is given, its 95% confidence limits follow in brackets. Cox multivariate analyses were made using program 2L in the BMDP package and a step-up procedure to identify the important independent prognostic factors. The statistical significance of any factor could be estimated from the ratio ($Z$) of the coefficient ($\beta$) for that factor to its standard error, $Z$ being treated as a normal deviate, i.e. $Z > 1.96$ corresponding to $P < 0.05$.

### RESULTS

Table 1 shows the distribution of the six social class categories of the 2455 patients studied. Forty-eight per cent fell into the non-manual categories compared with the 38% that would be predicted from the 1971 census (OPCS, 1975) for a general population of the same age distribution and having the same proportion of single women as our study group. This difference is not unexpected as the incidence of breast cancer has been shown to be greater in the better-off social classes (Adami et al, 1990).

In Table 2 patients in non-manual and manual categories are compared for possible important prognostic factors. The only statistically significant difference is the higher percentage of single women in the non-manual group. The other differences tend to favour the non-manual group, e.g. slightly more patients presenting with smaller tumours in stage I and slightly fewer patients with grade III tumours, but none of these differences is significant.

Table 3 shows a similar comparison for patients divided by weight at 60 kg. There are several highly significant differences, i.e. heavier patients tend to be older and more likely to be post-menopausal, to present with larger tumours and yet to be in stage I. There appears to be no correlation between weight and tumour grade.

Figure 1 shows the survival curves for the manual and non-manual groups. Although the non-manual group has fared better

 © Cancer Research Campaign 1997

| | | | | | | |
|---|---|---|---|---|---|---|
| No. at | 1130 | 817 | 595 | 448 | 141 | ≤ 60 kg |
| risk | 1325 | 918 | 618 | 437 | 139 | > 60 kg |

**Figure 2** Survival curves for patients divided by weight; all deaths counted as events. ●, ≤ 60 kg; ○, > 60 kg throughout the follow-up period, the difference is not statistically significant [RR = 1.07 (0.97–1.19); $P$ = 0.12]. Figure 2 shows a similar comparison by weight. Beyond 4 years, the curves are markedly divergent; the patients weighing less than or equal to 60 kg doing better [RR = 1.20 (1.08–1.33), $P$ = 0.002].

When the comparison is stratified by menopausal status (post-menopausal *vs* pre- and peri-menopausal combined), the effect is seen to be confined to the post-menopausal patients [RR = 1.27 (1.12–1.45), $P$ = 0.002] (Figure 3). The interaction is statistically significant ($P$ = 0.03). However, when the post-menopausal patients were further stratified by clinical stage, the effect of weight was apparent in both stages ($P$ = 0.006 in stage I, $P$ = 0.03 in stage II).

It is known that obesity affects overall mortality, and some of the increased mortality associated with weight in our series could be related to causes other than breast cancer. A careful study of the causes of death in this series after 5 years' follow-up has already been made and reported (Houghton et al, 1994). Figure 4A shows the effect of weight upon breast cancer deaths after 5 years in post-menopausal patients. Although there is little difference between the curves for patients weighing 61–70 kg and > 70 kg, there is a progressive increase of mortality with increase in weight from ≤ 50 kg to > 60 kg: ($\chi^2$ for trend computed for the four curves = 19.3, $P$ < 0.0001). The RR (> 60 kg *vs* ≤ 60 kg) is 1.68 (95% CI 1.33–2.12, $P$ <0.0001). By contrast, when only deaths from other causes are counted as events (Figure 4B), weight seems to have very little effect [RR = 1.03 (0.77–1.39), $P$ = 0.83], although the confidence limits mean that a possible real difference in non-breast cancer deaths cannot be excluded.

Cox multivariate analyses were made on the post-menopausal data with the results shown in Table 4. For these analyses, age was entered in years, weight in kilograms, tumour size in centimetres, clinical stage as 1 or 2, socioeconomic status as non-manual = 1, manual = 2 and treatment allocation as WP = 1, DXT = 2. For all deaths over the whole period, the effect of weight was confirmed – age, tumour size, stage and weight being selected as significant prognostic factors. The $P$-value for socioeconomic status to enter the model was 0.19. For deaths from breast cancer after 5 years, weight was selected with age and tumour size as significant factors. The $P$-value for socioeconomic status to enter the model was 0.91. For deaths from other causes, only age and treatment policy were selected – the $P$-value for socioeconomic status to be entered being 0.17. Similar analyses on the subset in which tumour grade was known showed the same significant effect of weight on deaths from breast cancer.

| | | | | | | | | | | | |
|---|---|---|---|---|---|---|---|---|---|---|---|
| No. at | 524 | 387 | 291 | 228 | 84 | 606 | 430 | 304 | 220 | 57 | ≤ 60 kg |
| risk | 456 | 337 | 255 | 203 | 73 | 869 | 581 | 363 | 234 | 66 | > 60 kg |

**Figure 3** Survival curves for patients divided by weight; all deaths counted as events. ●, ≤ 60 kg; ○, > 60 kg. (**A**) Pre- and peri-menopausal. (**B**) Post-menopausal

**Table 4** Results of Cox multivariate analyses on post-menopausal patients

| Follow up period | Event | Factors selected | β | Z-value | P-value |
|---|---|---|---|---|---|
| 0 + years | All deaths | Weight (kg) | 0.0106 | 3.70 | 0.0003 |
| | | Age (years) | 0.0236 | 4.40 | <0.0001 |
| | | Size (cm) | 0.1496 | 5.35 | <0.0001 |
| | | Stage | 0.2481 | 3.34 | 0.0011 |
| 5 + years | Death from breast cancer | Weight (kg) | 0.0225 | 4.75 | <0.0001 |
| | | Age (years) | 0.0211 | 2.31 | 0.021 |
| | | Size (cm) | 0.0952 | 2.00 | 0.045 |
| | Death from other causes | Age (years) | 0.0849 | 6.21 | <0.0001 |
| | | Treatment | 0.3350 | 2.21 | 0.027 |

**Figure 4** Survival curves for post-menopausal patients after 5 years. (**A**) Deaths from breast cancer. ●, ≤ 50 kg; □, 51–60 kg; ■, 61–70 kg; ○, > 70 kg. (**B**) Deaths from other causes. ●, ≤ 60 kg; ○, > 60 kg

### DISCUSSION

In this particular group of breast cancer patients, socioeconomic status as measured by the OPCS manual and non-manual categories does not appear to have been an important prognostic factor. Although the trend shown in Figure 1 is in the same direction as that found in previous studies, i.e. patients from manual occupations doing worse, the difference was not statistically significant, nor was it found to be so in any Cox analyses. This may be because our patient population was comparatively homogeneous in clinical presentation, primary treatment and follow-up. All patients had operable early breast cancer which was treated surgically by simple mastectomy, the only variation in primary treatment being the addition or omission of post-operative radiation. However, one of the American studies (Gordon et al, 1992) that found a significant effect of socioeconomic status was also conducted on patients entered for two clinical trials who were operable and treated with a modified radical mastectomy with or without adjuvant therapy and would, therefore, be similar to ours in homogeneity of clinical presentation and primary treatment. The range of the follow-up period in the American study was 5–16 years, compared with 16–21 years in our series which could, perhaps, influence our finding if the effect of socioeconomic status was confined to the early years after first treatment. This does not seem to be the case as a Cox analysis of all deaths in the first 5 years did not select socioeconomic status for the model; the *P*-value for it to be entered was 0.18.

The information on occupation recorded in our study was often very limited so that some errors in allocation to the six OPCS classes could well have occurred. But the decision whether a patient should be allocated to the manual or non-manual group was often less in doubt than the allocation to a particular social class. The final non-manual – manual ratio in our series is reasonable when compared with that in the general population (Table 1).

On the other hand, it is surprising that we found no relationship between weight and social class (Table 3) as there is evidence from many other studies that there is a strong association between obesity and social class, with the tendency for those in lower social classes and, in particular, women of these classes to be more obese (Stunkard and Sorensen, 1993; Carpenter and Bartley, 1994). Occupation can be only a very crude measure of differences in life style and deprivation. Other measures, such as area of residence, housing tenure or length of education, have often been used and may be better for differentiation. We conclude, therefore, that although our results do not provide any convincing evidence that social class affects prognosis, they cannot be taken as a strong argument against such a proposition.

The effect of weight on prognosis in our series is quite clear. In post-menopausal patients the risk of dying is increased with increased body weight, whereas in pre- and perimenopausal patients weight had no effect on mortality. The effect in post-menopausal patients was brought about by an increase in deaths from breast cancer rather than from other causes. This result is consistent with the review of previous studies by Goodwin and Boyd (1990). The relative risk (> 60 kg *vs* ≤ 60 kg) of dying from breast cancer after 5 years, derived from a multivariate analysis, for originally post-menopausal patients in our series was 1.59 (1.25–2.01), which is similar to the value derived for the effect of tumour size (> 2 cm *vs* ≤ 2 cm), i.e. RR = 1.32 (1.02–1.71).

 © Cancer Research Campaign 1997

The implication of this result is that weight should be included as an independent prognostic factor when comparing the survival outcome of post-menopausal patients treated by different strategies or when deriving prognostic indices for allocating patients to different risk categories. Korn and Simon (1990) point out that many prognostic models are not highly predictive even though the covariates in the model are highly statistically significant. In a group of breast cancer patients followed up for a median time of about 12 years, Schemper (1993) found that tumour grade and lymph node status, generally regarded as two of the most important prognostic factors, only explained 20% of the observed variability in outcome. Additional prognostic factors as a means of improving the prediction of prognosis are therefore desirable, and weight would appear to be an obvious candidate to be investigated in post-menopausal women treated for early breast cancer.

### ACKNOWLEDGEMENTS

We thank all those who took part in the trial and the Cancer Research Campaign for its continued financial support. We are most grateful to Ms B Wharram for a thorough reassessment of the occupational data and the derived social class categories.

### REFERENCES

Adami H-O, Adams G, Boyle P, Ewertz M, Lee NC, Lund E, Miller AB, Olsson H, Steel M, Trichopoulos D and Tulinius H (1990) Breast cancer etiology. *Int J Cancer* **5** (suppl.): 22–39

Blane D, Davey Smith G and Bartley M (1990) Social class differences in years of potential life lost: size, trends and principal causes. *Br Med J* **301**: 429–432

BMDP (1990) *Statistical Software Manual*. Dixon WJ (ed.), University of California Press: Berkeley

Cancer Research Campaign Working Party (1980) Cancer Research Campaign (King's/Cambridge) trial for early breast cancer. *Lancet* **2**: 55–60

Carpenter L and Bartley M (1994) Fat, female, and poor. *Lancet* **344**: 1715–1716

Elston CW, Gresham GA, Rao GS, Zebro T, Haybittle JL, Houghton J and Kearney G (1982) Cancer Research Campaign (King's/Cambridge) trial for early breast cancer: clinicopathological aspects. *Br J Cancer* **45**: 655–669

Ewertz M, Gillanders S, Meyer L and Zedeler K (1991) Survival of breast cancer patients in relation to factors which affect the risk of developing breast cancer. *Int J Cancer* **49**: 526–530

Goodwin PJ and Boyd NF (1990) Body size and breast cancer prognosis: a critical review of the evidence. *Breast Cancer Res Treat* **16**: 205–214

Gordon MH, Crowe JP, Brumberg DJ and Berger NA (1992) Socioeconomic factors and race in breast cancer recurrence and survival. *Am J Epidemiol* **135**: 609–618

Haybittle JL, Blamey RW, Elston CW, Johnson J, Doyle PJ, Campbell FC, Nicholson RI and Griffiths K (1982) A prognostic index in primary breast cancer. *Br J Cancer* **45**: 361–366

Haybittle JL, Brinkley D, Houghton J, A'Hern RP and Baum M (1989) Post-operative radiotherapy and late mortality: evidence from the Cancer Research Campaign trial for early breast cancer. *Br Med J* **298**: 1611–1614

Houghton J, Baum M and Haybittle JL (1994) The role of radiotherapy following total mastectomy in patients with early breast cancer. *World J Surg* **18**: 117–122

Katoh A, Watzlaf VJM and D'Amico F (1994) An examination of obesity and breast cancer survival in post-menopausal women. *Br J Cancer* **70**: 928–933

Korn EL and Simon R (1990) Measures of explained variation for survival data. *Stat Med* **9**: 487–503

OPCS. (1975) *Census 1971 Great Britain. Economic Activity. Part IV. (10% sample)*. HMSO: London

Schemper M (1993) The relative importance of prognostic factors in studies of survival. *Stat Med* **12**: 2377–2382

Schrijvers CTM and Mackenbach JP (1994) Cancer patient survival by socioeconomic status in seven countries: a review for six common cancer sites. *J Epidemiol Comm Health* **48**: 441–446

Schrijvers CTM, Mackenbach JP, Lutz J-M, Quinn MJ and Coleman MP (1995) Deprivation and survival from breast cancer. *Br J Cancer* **72**: 738–743

Stunkard AJ and Sorensen TIA (1993) Obesity and socioeconomic status – a complex relation. *NEng J Med* **329**: 1036–1037

© Cancer Research Campaign 1997